# Diurnal variation of NMDA receptor expression in the rat cerebral cortex is associated with traumatic brain injury damage

**DOI:** 10.1186/s13104-018-3258-0

**Published:** 2018-02-21

**Authors:** Francisco Estrada-Rojo, Julio Morales-Gomez, Elvia Coballase-Urrutia, Marina Martinez-Vargas, Luz Navarro

**Affiliations:** 1 Departamento de Fisiología, Facultad de Medicina, Universidad Nacional Autonoma de Mexico, Mexico City, Mexico; 2 Programa de Posgrado en Ciencias Biologicas, Universidad Nacional Autonoma de México, Mexico City, Mexico; 3 Laboratorio de Neurociencias, Instituto Nacional de Pediatria, Mexico City, Mexico

**Keywords:** Excitotoxicity, Circadian rhythm, Glutamate receptor

## Abstract

**Objective:**

Data from our laboratory suggest that recovery from a traumatic brain injury depends on the time of day at which it occurred. In this study, we examined whether traumatic brain injury -induced damage is related to circadian variation in *N*‐methyl‐d‐aspartate receptor expression in rat cortex.

**Results:**

We confirmed that traumatic brain injury recovery depended on the time of day at which the damage occurred. We also found that motor cortex *N*‐methyl‐d‐aspartate receptor subunit NR1 expression exhibited diurnal variation in both control and traumatic brain injury-subjected rats. However, this rhythm is more pronounced in traumatic brain injury—subjected rats, with minimum expression in those injured during nighttime hours. These findings suggest that traumatic brain injury occurrence times should be considered in future clinical studies and when designing neuroprotective strategies for patients.

## Introduction

Traumatic brain injuries (TBIs) are among the most important contemporary health problems. TBIs are responsible for longer periods of disability than any other cause [1].

The initial damage from a TBI range from contusion to skull fracture, hemorrhage, and diffuse axonal injury. The TBI then triggers secondary injuries that are mainly mediated by excitotoxicity resulting from substantial neuronal Ca^2+^ entry. This Ca^2+^ entry in turn results from glutamate binding to *N*‐methyl‐d‐aspartate receptors (NMDARs), which contribute to several dysfunctions, such as hypoperfusion [2], edema [3, 4], excitotoxicity [5], and cognitive deficits [6].

NMDARs are heteromers composed of two subunits types NR1 and NR2 [7, 8]. The NR1 subunits play the main functional role and are involved in apoptosis following injuries like ischemia or TBI [9].

We previously showed that TBI-induced damage depends on the time of day at which the TBI occurs [10, 11]. This may arise from temporal variation in NMDAR expression. Although several articles describe diurnal variations for numerous receptors [12], very few exist for NMDARs, and most of them in suprachiasmatic nuclei [13, 14], but there are reports in other tissues like hippocampus [15].

We are interested in the motor cortex because it is involved in movement initiation and the suppression of unwanted movements [16]. However, there is no data regarding diurnal variations in NMDAR expression within this structure. If such variations exist, then they might be associated with variations in TBI-triggered excitotoxicity.

Here, we explore the possible association between diurnal variation in rat cerebral cortex NMDAR expression and behavioral recovery from TBIs induced at different hours.

## Main text

### Methods

#### Subjects

Male Wistar rats (250–300 g) were maintained under controlled temperature and dark–light cycle (12:12 h; lights on at 08:00 h) with food and water ad libitum. All animal experiments were approved by the local ethics committee (protocol 128-2009, Facultad de Medicina, UNAM) and conducted according to official guidelines (NOM-062-ZOO-1999).

#### TBI

Rats were anesthetized with chloral hydrate [Riedel–de Haen, Germany] (350 mg/kg, i.p.) and subjected to TBIs with a modified closed-skull weight-drop injury protocol [17, 18]. Severe TBIs were induced on the exposed skull over the motor cortex (L:1.4, A:2) located with a stereotaxic device as previously described [11]. It consists of a pneumatic piston, which can be controlled in terms of the firing pressure and distance, therefore the magnitude of the impact is perfectly controlled. Previous trials in our laboratory allow us to establish with precision that our damage is similar in each subject. This model also reproduces focal damage [19], epidural hematoma and skull fracture (with or without brain damage) [20] and acute post-traumatic hemorrhage associated with severe TBIs in humans [21]. Furthermore, MRI studies have shown that this model accurately represents the clinical conditions that occur in closed skull lesions in humans, such as those occurring in falls or motor vehicle accidents [22].

#### Neurological behavior

We used a 21-point behavioral-neurological scale [23] to evaluate neurological status in control rats and TBI-subjected rats 24 h after model induction. Although this scale was designed to evaluate damage caused by cerebral ischemia, many pathophysiological pathways are reportedly activated in both forms of brain damage [24], and we have previously used this scale to investigate TBI-induced neurological damage [10, 11, 25, 26].

#### Diurnal variations

**Fig. 1:**
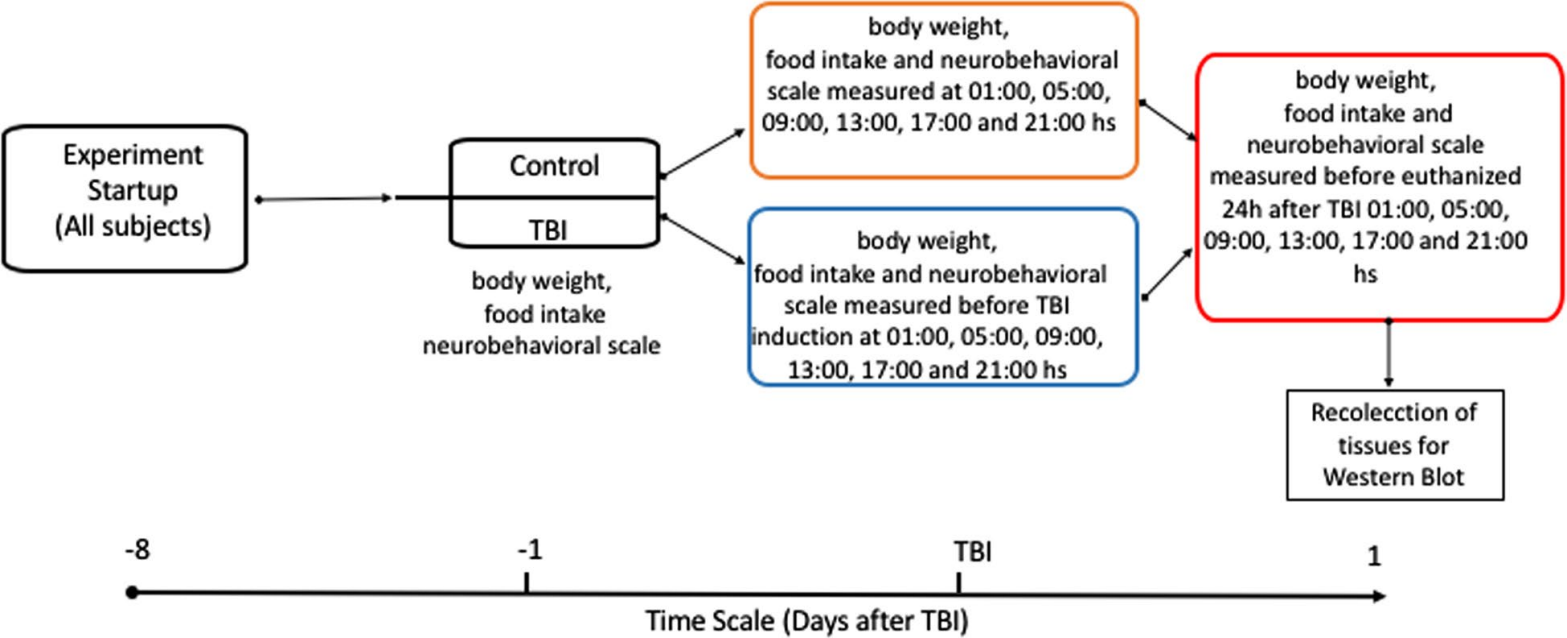
Experimental design

Control rats were subjected to neurobehavioral analysis before being deeply anesthetized with sodium pentobarbital [Pisa, México] (40 mg/kg, i.p.) and euthanized at different hours of the day (01:00, 05:00, 09:00, 13:00, 17:00, or 21:00 h) that we have previously used to analyze the diurnal variations of other receptors [11]. The motor cortex was dissected from each rat and stored at − 70 °C (Fig. 1).

We used another group of rats to analyze the effects of TBI on behavioral variables. These rats were anesthetized with chloral hydrate and subjected to TBI at different times (01:00, 05:00, 09:00, 13:00, 17:00, or 21:00 h). We quantified mortality immediately after TBI induction. After 24 h, we measured bodyweights and used a neurobehavioral scale to assess motor skill behaviors. The rats were deeply anesthetized with sodium pentobarbital and euthanized. The ipsilateral motor cortex was dissected and stored at − 70 °C (Fig. 1).

#### Western blotting

The motor cortex was homogenized with phosphate-buffered saline (PBS) [JT Baker, México] and protease inhibitors [Sigma-Aldrich, USA], and centrifuged (600*g*, 10 min, 4 °C). The supernatant was centrifuged again (39,000*g*, 15 min, 4 °C). Electrophoresis through a 7% analytical sodium dodecyl sulfate [JT Baker, México] -polyacrylamide [Merck, México] gel was performed. The resuspended tissue homogenates (60 μg of protein) were mixed with Laemmli buffer (1:1 ratio), heated (95 °C, 5 min) and loaded into a 0.75-mm-thick gel. The samples were electrophoresed (150 V, 2 h), and electroblotted (100 V, 1 h, 4 °C) onto a nitrocellulose membrane (GE Healthcare Life Sciences, Chicago, IL). The membrane was stained with Ponceau S [Sigma-Aldrich, USA], and cut into two pieces. One piece was used to analyze the glyceraldehyde 3-phosphate dehydrogenase (GAPDH) levels, and the other to analyze NR1 levels. The membrane was washed and incubated with 3% PBS-Tween, 10 or 20% nonfat dry milk, and 2 or 6% normal goat serum [Sigma-Aldrich, USA], for 30 min at room temperature. It was then incubated with anti-GAPDH (1:2000; Santa Cruz Biotechnology [sc166545], Dallas, TX) or anti-NR1 (1:1000; Abcam [ab17345], Cambridge, UK) antibodies overnight at 4 °C. The blot was thrice washed with PBS-Tween for 5 min, incubated for 1 h at room temperature with goat anti-rabbit immunoglobulin G horseradish peroxidase conjugate [Sigma-Aldrich, USA] (1:2000), and developed with diaminobenzidine [Sigma-Aldrich, USA] (0.5 mg/mL in PBS plus 0.009% H_2_O_2_). The band density was analyzed with Quantity One software (Bio-Rad Laboratories, Hercules, CA).

#### Statistical analysis

The results are reported as mean values ± standard errors of the mean (SEM). Statistical significance was assessed with two-way analysis of variance and Tukey’s multiple comparisons corrections for bodyweights and NR1 expression, Kruskal–Wallis and Mann–Whitney U tests for neurological scores, and Chi square tests for survival data. Statistical significance was defined as P < 0.05.

## Results

**Fig. 2:**
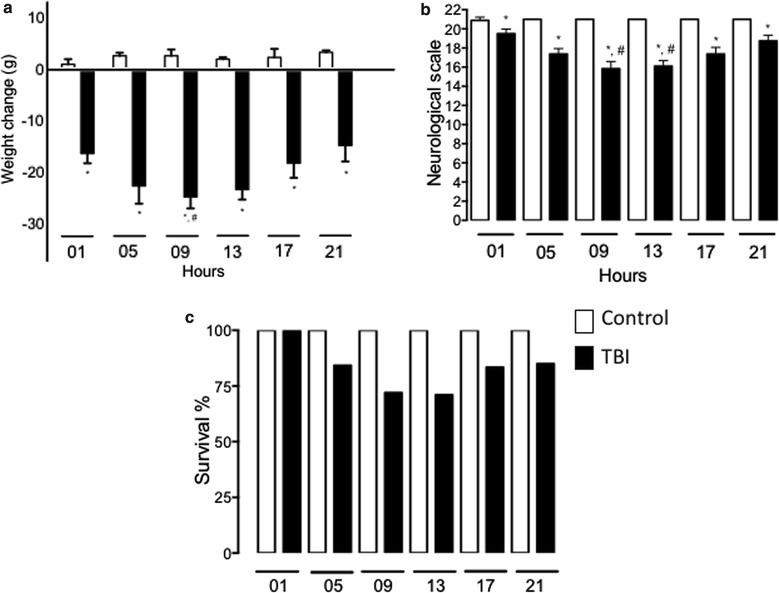
Effects of TBI induction time on bodyweight, neurobehavioral damage, and survival. **a** Bars represent the means ± standard errors of bodyweight losses 24 h after TBI induction at different times of day; *P < 0.05 vs control group at the same hour, ^#^P < 0.05 vs TBI group at 21:00 h. **b** Bars represent the means ± standard errors of neurobehavioral test scores obtained 24 h after TBI induction at different times of day; *P < 0.05 vs control group at the same hour, ^#^P < 0.05 vs TBI group at 01:00 and 21:00 h. **c** Bars represent the survival rates observed over a 24-h period following TBI induction at different times of day. n = 7–10 per timepoint in each group

Figure 2a shows that 24 h after the induction of the TBI, bodyweight losses significantly varied depending on the TBI induction time (F_1,107_ = 287.0, P < 0.0001).

Figure 2b shows the neurobehavioral scale findings. The rats with TBIs induced during daytime hours had lower scores than did those with TBIs induced at night (Kruskal–Wallis = 64.52, P < 0.0001).

Finally, Fig. 2c shows the survival rates following TBIs induced at different times. The survival rate was 100% for TBIs induced at 01:00 h but 71% for those induced at 13:00 h ($\upchi^{ 2 }_5 = 3. 6 2 8$, P = 0.6041). TBIs induced during daytime hours tended to cause greater damage than those induced at night did.

**Fig. 3:**
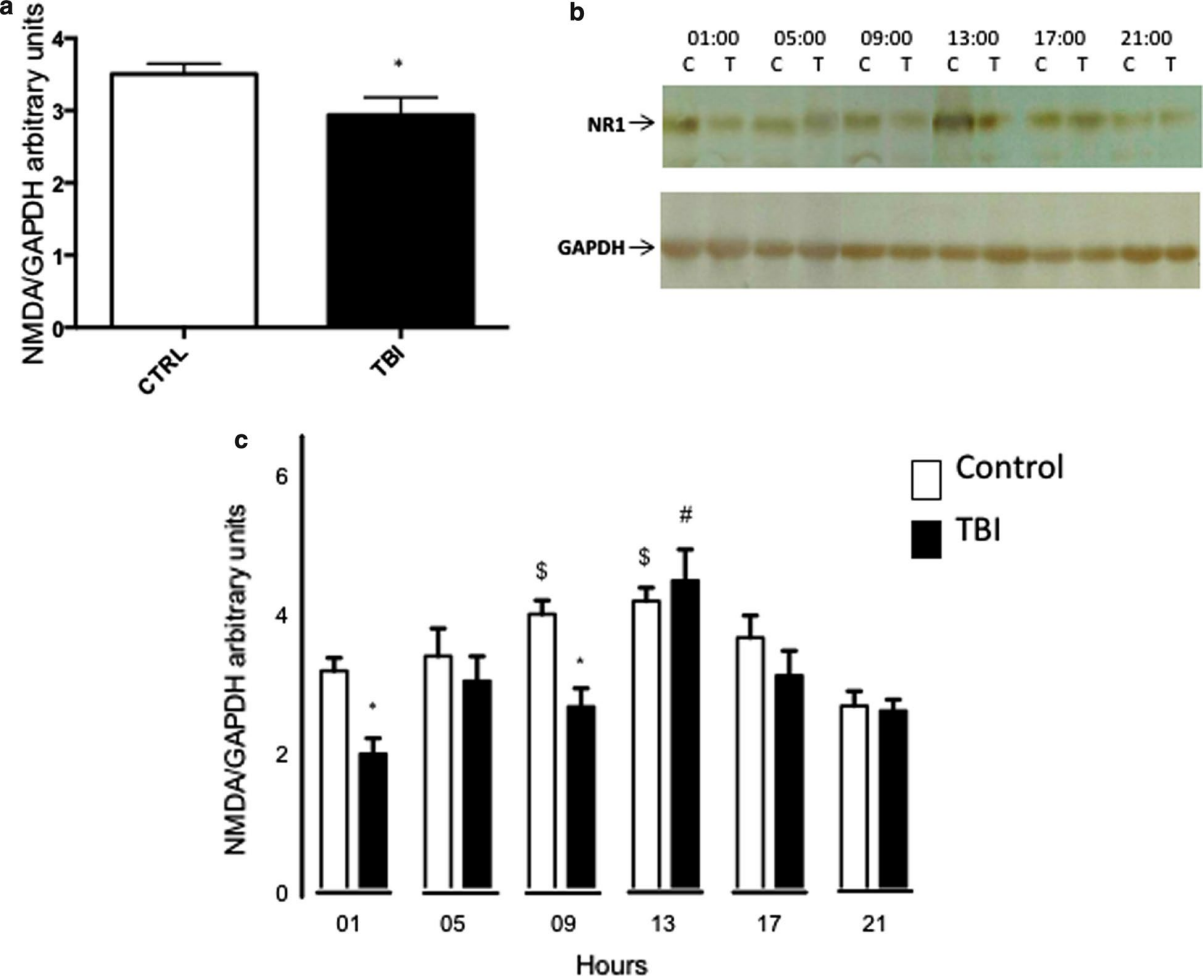
Effects of TBI and TBI induction time on NMDAR subunit NR1 expression in the rat cerebral cortex. **a** Data considering all the controls as a single group and all the experimental ones as another group. Bars represent means ± standard errors. *P < 0.05 vs control. **b** Representative Western immunoblotting of NR1 and GAPDH in motor cortex. **c** Data considering TBI induction time. Bars represent means ± standard errors. *P < 0.05 vs control group at the same hour; ^$^P < 0.05 vs control group at 21:00 h; ^#^P < 0.05 vs TBI group at 01:00 and 21:00 h. n = 7–9 per timepoint in each group

Figure 3 shows the observed expression of NMDAR subunit NR1 in the motor cortex. Figure 3a includes the results for all control and TBI-subjected rats. We found that TBIs induced a significant reduction of NMDAR expression in the motor cortex (t_104_ = 2.130, P = 0.0356). Nevertheless, this reduction depended on the TBI induction time. We found significant effects of experimental group (F_1,94_ = 10.88, P = 0.0014), TBI induction time (F_5,94_ = 10.21, P < 0.0001), and the two factors’ interaction (F_5,94_ = 2.540, P = 0.0334) (Fig. 3b, c). In control rats, we found that NR1 expression was greater at 09:00 and 13:00 h than at 21:00 h, whereas in TBI-subjected rats, NR1 expression was greater at 13:00 h than at 01:00, 09:00, and 21:00 h. Notably, TBIs induced at 01:00 h or 09:00 h reduced NR1 expression, but those induced at other times did not.

## Discussion

TBIs are a major health problem and affect millions of people worldwide every year. They are associated with short- and long-term damage in several domains, including physical, cognitive, metabolic, and behavioral problems, which depend on the severity of the damage [27, 28].

TBIs induce abnormal homeostasis in areas of secondary damage. The influx of glutamate into the extracellular space is immediate and can last several minutes. This produces excessive NMDAR activation, which in turn produces excessive and uncontrolled entry of Na^+^ and Ca^2+^ into neurons [28–30]. This Ca^2+^ influx interferes with mitochondrial oxidative phosphorylation and produces oxidative stress and apoptosis. This effect is amplified by neuronal hyperexcitability, which exacerbates glutamate release and produces a vicious circle involving generalized excitotoxicity [31–36].

The excessive glutamate release causes astrocytes to recapture glutamate via the glutamate transporter GLT-1 [37], but persistently high extracellular glutamate concentrations can cause down-regulated of GLT1 and intracellular Na^+^ overload reverses glutamate transport. This causes glutamate efflux from the astrocytes, increasing excitotoxic damage [38, 39].

Moreover, NMDAR-mediated Ca^2+^ loading is more neurotoxic than equivalent Ca^2+^ loading mediated by other mechanisms [40]. Hardingham [41] suggests that neuronal responses by NMDA activation follow a bell-shaped curve in which excessively high and excessively low activity are both potentially harmful.

In this study, we hypothesized that the dependence of post-TBI behavioral recovery on TBI induction time is related to diurnal variation in rat motor cortex NMDAR expression. Our results support our initial hypothesis, we found that a TBI induced deterioration in an animal’s general condition. This manifested as decreased bodyweight, as reported by other authors [42, 43]. We also verified that post-TBI deterioration is observable on a neurobehavioral scale. This finding and the survival rates data corroborate our previous findings that showed that post-TBI recovery depends on the time of day at which the TBI occurs. This time-dependent recovery must respond to some rhythmic event that modulates the post-TBI damage. We proposed that this rhythmic event could be diurnal variations in the glutamatergic system. Given that excitotoxicity depends on NMDAR activation, we evaluated NMDAR expression in the area of damage. We propose that a greater expression of NMDAR produces greater influx of Ca^2+^ and therefore greater excitotoxicity, the opposite should happen in the hours with low expression levels and therefore should be less post-TBI damage. We therefore analyzed the expression of the NR1 subunit since, as indicated above, it is essential in the functioning of the NMDAR [7, 8, 44].

Several authors have shown that after a traumatic event there are changes in the expression of NMDAR, these effects were manifested from the first minutes until a week later and in various regions such as frontoparietal cortex and hippocampus, the expression also varies depending on the area of impact (decrease) or penumbra (increase) [45, 46].

With respect to the NR1 subunit, some authors found no variation in cortex and hippocampus between 1 and 7 days post-TBI [7, 8, 47] while others only saw a transient change in the hippocampus [48].

We found that the effect of TBI on NR1 expression depended on the TBI induction time. Moreover, our data show that the extent of TBI-induced damage also depended on the TBI induction time. TBIs caused less damage if they occurred during nighttime hours. Such nighttime TBIs were associated with decreased NMDAR expression and lower extracellular glutamate levels in the motor cortex as reported by Dash [49], which support Hardingham’s [41] proposal.

Numerous animal studies have shown that NMDAR antagonists can mitigate TBI-induced damage. However, clinical trials have found that such drugs have no benefits and even have detrimental effects [50]. Several authors have discussed the causes of these failures in humans and have suggested that they include trial design, human population heterogeneity, and inadequate dose ranges [51, 52]. We believe that another factor worth considering is the TBI occurrence time, since NMDAR expression depends on this variable.

## Limitations

A TBI induces behavioral damage.

The extent of TBI-induced damage depends on the time of day the TBI is induced.

NMDAR activation-induced excitotoxicity is a primary mechanism of TBI damage.

NMDAR—subunit NR1 expression in cerebral cortex depends on the time of day the TBI is induced.

The minimal expression of NMDA—subunit NR1 is associated with a lesser damage caused by TBI.

The diurnal variation of NMDAR functionality in cerebral cortex has not been analyzed.

## Abbreviations

GAPDH: glyceraldehyde 3-phosphate dehydrogenase
NMDAR: *N*‐methyl‐d‐aspartate receptor
PBS: phosphate-buffered saline
TBI: traumatic brain injury
